# Associations Between Malignant Tumors and Alzheimer's Disease: A Cross‐Sectional Study

**DOI:** 10.1002/brb3.71066

**Published:** 2025-11-14

**Authors:** Yun Zhang, Yihao Wang, Yang Zhao, Yan Xin, Lei Zhang, Peipei Shan, Youzhuang Zhu

**Affiliations:** ^1^ Department of Anesthesiology The Affiliated Hospital of Qingdao University Qingdao Shandong Province China; ^2^ Department of Pain The Affiliated Hospital of Qingdao University Qingdao Shandong Province China; ^3^ Department of Anesthesiology Qingdao Municipal Hospital Qingdao Shandong Province China; ^4^ Institute of Translational Medicine The Affiliated Hospital of Qingdao University, College of Medicine, Qingdao University Qingdao Shandong Province China

**Keywords:** alzheimer's disease, cognitive function, malignant tumors, neurofilament light chain

## Abstract

**Introduction:**

Although there is potential overlap in the pathological and physiological mechanisms underlying malignant tumors and Alzheimer's disease (AD), a significant gap remains in the epidemiological evidence supporting their association. We used data from the National Health and Nutrition Examination Survey (NHANES) to elucidate the epidemiological relationship between malignant tumors and AD.

**Methods:**

We analyzed data from NHANES from 2013 to March 2020, prior to the COVID‐19 pandemic, including 9274 participants aged ≥ 20 years. Multivariate logistic regression was used to explore the link among malignant tumors, tumor types, and AD risk. Sensitivity analyses were performed to evaluate the robustness of the association with neurofilament light chain levels, composite cognitive function scores, and different analytic populations. Additionally, subgroup analyses were performed to explore the association between malignancy and AD risk in specific subpopulations.

**Results:**

Among the 9274 participants included in the study, 1432 were identified as having malignant tumors. There was a significant association between malignant tumors and AD [adjusted odds ratio (aOR): 1.55; 95% confidence interval (CI): 1.27–1.89; *p* < 0.001]. The risk of AD was higher in patients with colorectal and prostate cancer compared to those with other malignancies. Sensitivity analysis of neurofilament light chain levels suggested that malignant tumors may contribute to the development of mild cognitive impairment (aOR: 1.52; 95% CI: 1.01–2.27; *p* = 0.044). Subgroup analysis revealed that AD risk following a malignant tumor diagnosis was higher in people < 65 years old and those with medium–high incomes.

**Conclusions:**

Malignant tumors may be significantly associated with increased AD risk, particularly in patients with colorectal and prostate cancer. Moreover, individuals < 65 years old and those with middle–high income had increased risk of developing AD following a malignancy. These findings underscore the significance of early AD detection and prevention in patients with a history of malignant tumors.

## Graphical abstract

This cross‐sectional study reveals that malignant tumors may be associated with AD risk, particularly in patients with colorectal and prostate cancer. The association is more pronounced among individuals <65 years old and those with middle–high income. Study findings confirm the necessity of AD diagnosis in patients with malignant tumors.

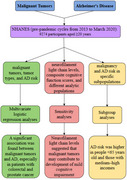

## 1 Introduction

Alzheimer's disease (AD) is a neurodegenerative condition characterized by progressive cognitive decline, with clinical signs encompassing a broad spectrum of neuropsychiatric manifestations (Scheltens et al. 2021). It is projected that by 2050, the global population of people with AD will increase from the current 57 million to 153 million (GBD 2019 Dementia Forecasting Collaborators 2022). As a major category of non‐communicable chronic diseases, AD ranks among the top five leading causes of mortality in individuals > 50 years old (Lin et al. 2024). Considering that AD is incurable, continuous expenses related to its care and treatment pose considerable challenges for both families and public health systems worldwide (Prince et al. 2016; Ji et al. 2024; Wimo et al. 2007).

The pathogenesis of malignant tumors and AD share overlapping features. In patients with malignant tumors, elevated levels of accumulated mutant p53 contribute to both tumorigenesis and tumor development (Oren and Rotter 2010; Liu et al. 2015). Conformational instability of p53 also is implicated in AD progression (Abate et al. 2020). Amyloid precursor protein, presenilin 1, and presenilin 2 are molecules involved in amyloid‐β metabolism in AD (Kametani and Hasegawa 2018). Primary neurons isolated from AD mouse models carrying amyloid precursor protein and presenilin 1 mutations exhibit impaired p53 activity (Abate et al. 2020; Sompol et al. 2008). Experiments using neuroblastoma‐differentiated neuron‐like cells with amyloid precursor protein overexpression show that unfolded p53 inhibits proteins involved in axon growth and synaptic plasticity (Tedeschi et al. 2009; Buizza et al. 2013). These findings suggest convergence in the mechanistic pathways underlying both malignant tumors and AD pathogenesis.

DNA methylation plays a dynamic role in modulating expression of oncogenes and tumor suppressor genes. This epigenetic reprogramming not only promotes tumor phenotypes but also contributes to the accumulation of epigenetic modifications associated with cognitive decline (Dor and Cedar 2018; Berson et al. 2018). The Wnt signaling pathway plays a vital role in controlling cell proliferation and maintaining neuronal balance. Dysregulation of this pathway exacerbates tumor metabolism and concurrently promotes AD features, such as tau hyperphosphorylation and cognitive decline (Pate et al. 2014; Mo et al. 2019; Kostes and Brafman 2023). Loss‐of‐function of canonical Wnt signaling not only induces AD‐like neuropathological changes in wild‐type mice but also accelerates pathological progression of AD mouse models (Tapia‐Rojas and Inestrosa 2018).

Oxidative stress serves as a common trigger in both malignant tumors and AD, generating reactive intermediates and disrupting redox signaling (Forman and Zhang 2021). Additionally, inflammatory cascades and abnormal cell cycle regulation are key pathophysiological connections between malignant tumors and AD. Tumor‐derived inflammatory mediators and oncometabolites might enter systemic circulation, compromising the blood–brain barrier and exacerbating neuronal injury—a plausible mechanism for acceleration of malignant tumor‐associated AD (Chevrier et al. 2017). Yet despite these complex interconnections, population‐level evidence from biomedical databases remains limited, emphasizing the pressing requirement for epidemiological research to further explore the association between malignant tumors and AD risk. Thus, we explored rich data from the National Health and Nutrition Examination Survey (NHANES) to better elucidate the epidemiological relationship between malignant tumors and AD.

## 2 Methods

### 2.1 Study Design and Setting

NHANES, a national project focused on health and nutrition assessment, is implemented by the US Centers for Disease Control and Prevention. The purpose of NHANES is to assess the health and nutritional conditions of adults and children. To guarantee that the sample is representative, NHANES uses a sampling approach that combines stratification and multiple stages of probability selection. All participants provided informed consent, and the study's protocol was approved by the Research Ethics Review Board of the US National Center for Health Statistics. Relevant data and additional information can be accessed through the official NHANES website. This study adheres to guidelines outlined in the Strengthening the Reporting of Observational Studies in Epidemiology statement.

### 2.2 Participants

This cross‐sectional study used data from the NHANES pre‐pandemic cycles from 2013 to March 2020. Of the 35,706 participants in these NHANES cycles, 9274 individuals aged ≥ 20 years were included in the final analysis. These participants had complete data on malignant tumor status, AD medication use, and other key covariates.

### 2.3 Variables

#### 2.3.1 Malignant Tumors

Individuals with malignant tumors were identified based on responses to the NHANES survey question: “Have you ever been told by a doctor or other health professional that you had cancer or a malignancy of any kind?” Trained interviewers administered the questionnaire in participants’ homes using a computer‐assisted personal interviewing system. This system incorporates built‐in consistency checks to minimize data entry errors and features online screens to help interviewers define key terms. Following data collection, NHANES field office staff reviewed interview records for accuracy and completeness. Malignant tumors types were classified according to participants' responses to the question: “What kind of cancer was it?”.

#### 2.3.2 Alzheimer's Disease (AD)

During NHANES household survey interviews, participants were asked about prescription medication use within the preceding 30 days. Since 2013, the US National Center for Health Statistics has used the International Classification of Diseases, Tenth Revision, Clinical Modification (ICD‐10‐CM) system to standardize classification of self‐reported medical conditions. Participant‐reported indications for prescription medications were mapped to ICD‐10‐CM codes for standardized reporting. Each ICD‐10‐CM code corresponds to a specific disease, symptom, or health status descriptor. For AD‐related medication use without subtype specification, the code G30.9 was assigned. AD diagnosis was confirmed if the patient was prescribed at least one of the following therapeutic agents: donepezil, galantamine, rivastigmine, memantine, or gabapentin.

We performed sensitivity analysis using three methods. First, neurofilament light chain (NfL) was examined as an objective biomarker for AD. NfL can detect pathological changes before the onset of clinical symptoms, supporting its role as a biomarker for the pre‐symptomatic stage of AD (Self and Holtzman 2023). A cohort study reported a threshold of 20.4 pg/mL NfL predicts the onset of mild cognitive impairment, the earliest clinical manifestation of AD (Albert et al. 2011; Mazzeo et al. 2024). Thus, we used ≥ 20.4 pg/mL NfL as a cutoff to identify individuals with mild cognitive impairment. Second, we used 2013–2014 NHANES data to explore the relationship between malignancy and composite cognitive function scores. We set the lowest quartile of three test scores as the threshold for diagnosing cognitive decline. Third, gabapentin can alleviate AD‐related behavioral changes and comorbid conditions (Moretti et al. 2003), but it is not a core therapy. Thus, we excluded patients who were defined as having AD only by taking gabapentin.

#### 2.3.3 Data Measurement

Demographic covariates included: (1) sex (male, female); (2) age (< 65 years, ≥ 65 years); (3) body mass index (BMI), analyzed both continuously and dichotomized according to World Health Organization criteria (non‐obese: <30 kg/m^2^; obese: ≥ 30 kg/m^2^); and (4) race, categorized as non‐Hispanic White, Black, Mexican American, or Other.

Socioeconomic factors comprised: (1) educational attainment, categorized as < high school (< 9th grade), high school (9th–12th grade), and ≥ college (> 12th grade); and (2) socioeconomic status, measured by the poverty‐income ratio, calculated as family income relative to the federal poverty threshold, with tertile stratification (low, medium, and high).

Health behavior covariates included: (1) physical activity, defined as routine ambulation (≥ 10 min continuous walking/bicycling for commuting, excluding occupational exertion); and (2) smoking status (never: < 100 lifetime cigarettes; former: ≥ 100 cigarettes but currently abstinent; current: ≥ 100 cigarettes with continued use).

Mental health and physical illnesses included: (1) depressive symptoms, assessed by self‐reported frequency of dysphoric mood episodes in the preceding two weeks (not at all, several days, most days, almost every day); (2) hypertension; (3) diabetes; (4) coronary heart disease (CHD); and (5) stroke.

Cognitive data were sourced from the NHANES database, encompassing tests such as the Consortium to Establish a Registry for Alzheimer's Disease (CERAD) Word Learning subtest (evaluating the capacity to acquire new verbal information), the Animal Fluency Test (AFT) (gauging categorical verbal fluency within executive function), and the Digit Symbol Substitution Test (DSST) (measuring processing speed, sustained attention, and working memory).

### 2.4 Statistical Analysis

Data handling and analysis were conducted using R (version 4.4.2) and Zstats (https://www.zstats.cn). Normally distributed data are expressed as mean ± standard deviation (SD), non‐normally distributed data as median and interquartile range (IQR), and categorical variables as frequency (n) and percentage (%). Baseline features were compared by applying the two‐sample t‐test, Mann–Whitney U test, or chi‐square test, as appropriate. Univariate logistic regression was performed to assess associations among malignant tumors, covariates, and AD, calculating odds ratio (OR) and 95% confidence interval (CI). Multivariate logistic regression was used to evaluate the association between malignant tumors and AD, yielding adjusted odds ratio (aOR) and 95% CI after adjusting for covariates. The sample was stratified into subgroups based on key characteristics, and interactions were assessed. Sensitivity analyses were conducted using single‐cycle NfL data and composite cognitive function scores, excluding patients taking gabapentin alone to assess the robustness of observed associations. Finally, malignant tumor type‐specific data for patients were extracted to further investigate the association between tumor types and AD. CERAD, AFT, and DSST lack standardized definitions for impaired cognitive function, so we adopted methods from previously published research (Chen et al. 2017). Scores were dichotomized using the respective minimum quartile thresholds: 22 for CERAD, 13 for AFT, and 34 for DSST. All statistical tests were two‐sided, with *p* < 0.05 considered statistically significant.

## 3 Results

### 3.1 Baseline Characteristics

**FIGURE 1:**
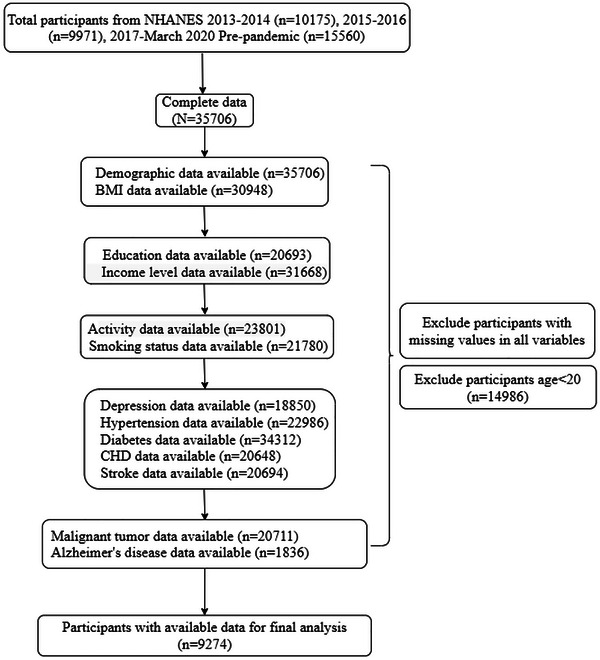
Flowchart of study participants. BMI, body mass index; CHD, coronary heart disease.

We analyzed NHANES data encompassing 9274 participants aged ≥ 20 years (Figure 1). Among the study population, 64.3% were < 65 years old, and 35.7% were ≥ 65 years old. Males constituted 44.4% of the population, while females accounted for 55.6%. There were 1432 (15.4%) cases of malignant tumors and 7842 (84.6%) cases of non‐malignant tumors. A total of 628 (6.77%) AD cases were identified. Significant differences were observed between participants with malignant and non‐malignant tumors in terms of age, sex, BMI, race, education, income level, activity, hypertension, CHD, stroke, and smoking status (Table 1).

**TABLE 1:** Baseline characteristics of patients with malignant or non‐malignant tumors.

Variables	All	Malignant tumors (n = 1432)	Non‐malignant tumors (n = 7842)	*P*
Age				< 0.001
< 65 years	5959 (64.3)	524 (36.6)	5435 (69.3)	
≥ 65 years	3315 (35.7)	908 (63.4)	2407 (30.7)	
Sex				0.014
Male	4117 (44.4)	678 (47.3)	3439 (43.9)	
Female	5157 (55.6)	754 (52.7)	4403 (56.1)	
Body mass index				< 0.001
< 30 kg/m^2^	4988 (53.8)	829 (57.9)	4159 (53)	
≥ 30 kg/m^2^	4286 (46.2)	603 (42.1)	3683 (47)	
Race				< 0.001
White	4187 (45.1)	933 (65.2)	3254 (41.5)	
Black	2099 (22.6)	219 (15.3)	1880 (24)	
Hispanic	988 (10.7)	90 (6.3)	898 (11.5)	
Other	2000 (21.6)	190 (13.3)	1810 (23.1)	
Education				< 0.001
< High school	721 (7.8)	79 (5.5)	642 (8.2)	
High school	3113 (33.6)	457 (31.9)	2656 (33.9)	
≥ College	5440 (58.7)	896 (62.6)	4544 (57.9)	
Income level				< 0.001
Low	2682 (28.9)	336 (23.5)	2346 (29.9)	
Medium	3491 (37.6)	577 (40.3)	2914 (37.2)	
High	3101 (33.4)	519 (36.2)	2582 (32.9)	
Depression				0.319
Not at all	6691 (72.1)	1054 (73.6)	5637 (71.9)	
Several days	1731 (18.7)	264 (18.4)	1467 (18.7)	
Most days	466 (5.0)	60 (4.2)	406 (5.2)	
Almost every day	386 (4.2)	54 (3.8)	332 (4.2)	
Physical activity				< 0.001
Yes	1825 (19.7)	217 (15.2)	1608 (20.5)	
No	7449 (80.3)	1215 (84.8)	6234 (79.5)	
Hypertension				<0.001
Yes	4284 (46.2)	765 (53.4)	3519 (44.9)	
No	4990 (53.8)	667 (46.6)	4323 (55.1)	
Diabetes				0.211
Yes	2135 (23.0)	348 (24.3)	1787 (22.8)	
No	7139 (77.0)	1084 (75.7)	6055 (77.2)	
Coronary heart disease				< 0.001
Yes	638 (6.9)	161 (11.2)	477 (6.1)	
No	8636 (93.1)	1271 (88.8)	7365 (93.9)	
Stroke				< 0.001
Yes	577 (6.2)	132 (9.2)	445 (5.7)	
No	8697 (93.8)	1300 (90.8)	7397 (94.3)	
Smoking status				< 0.001
Never	4932 (53.2)	647 (45.2)	4285 (54.6)	
Former	2746 (29.6)	579 (40.4)	2167 (27.6)	
Current	1596 (17.2)	206 (14.4)	1390 (17.7)	

Data are displayed as median (interquartile range) and frequency (percentage).

### 3.2 Risk Association between Malignant Tumors and AD

Univariate logistic regression identified age, BMI, race, education, income level, depression, activity, hypertension, diabetes, CHD, stroke, and smoking status as factors associated with AD (Supplementary Table 1). Further, we identified a significant association between malignant tumors and AD (OR: 1.55; 95% CI: 1.27–1.89; *p* < 0.001; Table 2). In multivariate logistic regression, malignant tumors remained significantly associated with AD after adjusting for covariates (aOR: 1.30; 95% CI: 1.05–1.61; *p* = 0.017).

**TABLE 2:** Risk association between malignant tumors and AD.

Variable	OR (95% CI)						
	n	Crude	*P*	Model 1	*P*	Model 2	*P*
Malignant tumors, n (%)							
No	7842	1 (reference)		1 (reference)		1 (reference)	
Yes	1432	1.55 (1.27–1.89)	< 0.001	1.34 (1.08–1.66)	0.007	1.30 (1.05–1.61)	0.017

*Note*: Model 1 adjusted for covariates including age, sex, body mass index, race, education, income level, depression, activity, and smoking status.

Model 2 adjusted for covariates including age, sex, body mass index, race, education, income level, depression, activity, smoking status, hypertension, diabetes, coronary heart disease, and stroke.

### 3.3 Association between Malignant Tumor Types and AD

Multivariate logistic regression analysis revealed a significant risk association between colorectal cancer and AD (aOR: 1.95, 95% CI: 1.08–3.52; *p* = 0.027) and between prostate cancer and AD (aOR: 1.67; 95% CI: 1.06–2.65; *p* = 0.028). No significant associations were observed between AD and other common malignancies (Table 3).

**TABLE 3:** Risk association of different types of malignant tumors with AD.

Variables	OR (95% CI)							
		n	Crude	*P*	Model 1	*P*	Model 2	*P*
Colorectal cancer, n (%)	No	9176	1 (reference)		1 (reference)		1 (reference)	
	Yes	98	2.32 (1.31–4.12)	0.004	1.89 (1.06–3.39)	0.032	1.95 (1.08–3.52)	0.027
Prostate cancer, n (%)	No	9039	1 (reference)		1 (reference)		1 (reference)	
	Yes	235	1.59 (1.03–2.44)	0.035	1.8 (1.15–2.83)	0.011	1.67 (1.06–2.65)	0.028
Lung cancer, n (%)	No	9223	1 (reference)		1 (reference)		1 (reference)	
	Yes	51	0.86 (0.27–2.77)	0.800	0.61 (0.19–2.01)	0.417	0.60 (0.18–1.99)	0.402
Stomach cancer, n (%)	No	9268	1 (reference)		1 (reference)		1 (reference)	
	Yes	6	0 (0–NA)	0.960	0 (0–NA)	0.956	0 (0–NA)	0.955
Liver cancer, n (%)	No	9263	1 (reference)		1 (reference)		1 (reference)	
	Yes	11	1.38 (0.18–10.78)	0.760	1.16 (0.15–9.28)	0.886	0.87 (0.11–6.94)	0.892
Thyroid cancer, n (%)	No	9253	1 (reference)		1 (reference)		1 (reference)	
	Yes	21	2.30 (0.68–7.83)	0.182	1.86 (0.53–6.56)	0.336	1.48 (0.41–5.3)	0.550
Cervical cancer, n (%)	No	9203	1 (reference)		1 (reference)		1 (reference)	
	Yes	71	2.28 (1.16–4.47)	0.017	2.1 (1.05–4.19)	0.036	1.93 (0.95–3.95)	0.070
Bladder cancer, n (%)	No	9232	1 (reference)		1 (reference)		1 (reference)	
	Yes	42	0 (0–NA)	0.954	0 (0–NA)	0.952	0 (0–NA)	0.950
Breast cancer*, n (%)	No	5062	1 (reference)		1 (reference)		1 (reference)	
	Yes	125	1.69 (0.92–3.09)	0.091	1.40 (0.74–2.64)	0.299	1.31 (0.69–2.48)	0.415

*Note*: *Only 2013–2014 and 2015–2016 cycle data are available for analysis.

**Abbreviations**: NA: not applicable.

Corresponding ICD‐10‐CM codes for malignant tumors in NHANES database: colorectal cancer (C18, C20), prostate cancer (C61), lung cancer (C34), stomach cancer (C16), liver cancer (C22), thyroid cancer (C73), cervical cancer (C53), bladder cancer (C67), breast cancer (C50).

Model 1 adjusted for covariates including age, sex, body mass index, race, education, income level, activity, and smoking status.

Model 2 adjusted for covariates including age, sex, body mass index, race, education, income level, activity, smoking status, hypertension, diabetes, coronary heart disease, and stroke.

### 3.4 Subgroup Analysis

**FIGURE 2:**
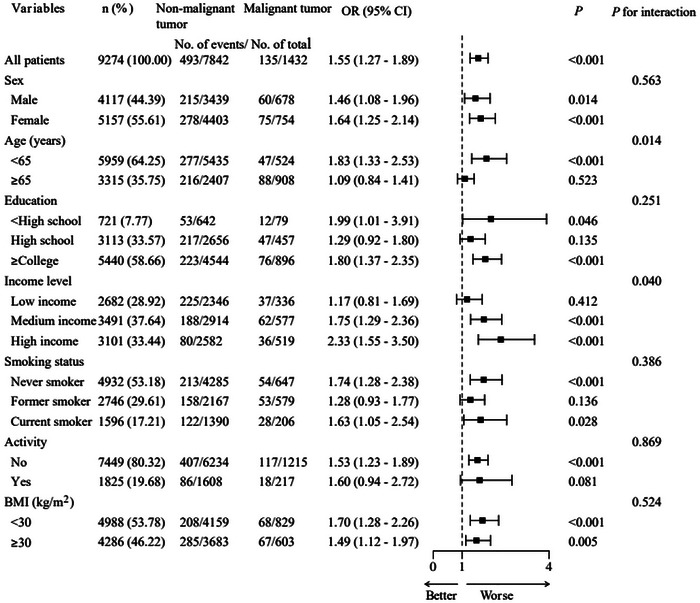
Subgroup analysis of the association between malignant tumors and Alzheimer's disease. BMI, body mass index; CI, 95% confidence interval; OR, odds ratio.

Subgroup analysis showed the association between malignant tumors and AD varied significantly by age and income level after adjusting for covariates. AD risk was higher in patients with malignant tumors aged < 65 years (aOR: 1.83; 95% CI: 1.33–2.53) compared to those aged ≥ 65 years (aOR: 1.09; 95% CI: 0.84–1.41; Figure 2). Among patients with malignant tumors, those in medium‐income (aOR: 1.75; 95% CI: 1.29–2.36) and high‐income (aOR: 2.33; 95% CI: 1.55–3.50) groups had a higher risk of developing AD compared to those in the low‐income group (aOR: 1.17; 95% CI: 0.81–1.69; Figure 2).

### 3.5 Sensitivity Analysis

Sensitivity analysis was conducted using NfL data from the 2013–2014 NHANES cycle, including 1770 individuals in the final analysis. Participants were stratified into malignant tumor (n = 138) and non‐malignant tumor (n = 1632) groups. Significant differences were observed between participants with malignant and non‐malignant tumors in terms of age, race, education, income level, hypertension, diabetes, stroke, and smoking status (Supplementary Table 2). Multivariable logistic regression analysis showed a significant association between malignant tumors and mild cognitive impairment after adjusting for covariates (aOR: 1.52; 95% CI: 1.01–2.27; *p* = 0.044; Table 4). None of the three scoring scales contributing to composite cognitive function individually showed a significant association between malignant tumors and cognitive function (Table 5).

**TABLE 4:** Sensitivity analysis between malignant tumors and mild cognitive impairment.

Variable	OR (95% CI)						
	n	Crude	*P*	Model 1	*P*	Model 2	*P*
Malignant tumors, n (%)							
No	1632	1 (reference)		1 (reference)		1 (reference)	
Yes	138	1.73 (1.18–2.52)	0.005	1.60 (1.08–2.38)	0.019	1.52 (1.01–2.27)	0.044

*Note*: Model 1 adjusted for gender, body mass index, race, education, income level, depression, activity, and smoking status.

Model 2 adjusted for gender, body mass index, race, education, income level, depression, activity, smoking status, hypertension, diabetes, coronary heart disease, and stroke.

**TABLE 5:** Sensitivity analysis between malignant tumors and composite cognitive function scores.

Variable	OR (95% CI)							
		n	Crude	*P*	Model 1	*P*	Model 2	*P*
Malignant tumors (CERAD)	No	1041	1 (reference)		1 (reference)		1 (reference)	
	Yes	296	0.96 (0.71–1.30)	0.792	1.05 (0.75–1.47)	0.762	1.06 (0.76–1.49)	0.720
Malignant tumors (AFT)	No	1041	1 (reference)		1 (reference)		1 (reference)	
	Yes	296	1.13 (0.82–1.56)	0.438	0.97 (0.69–1.38)	0.873	1.00 (0.70–1.42)	0.998
Malignant tumors (DSST)	No	1041	1 (reference)		1 (reference)		1 (reference)	
	Yes	296	1.47 (1.07–2.02)	0.019	1.33 (0.91–1.93)	0.138	1.42 (0.97–2.08)	0.074

**Abbreviations**: AFT: animal fluency test; CERAD: consortium to establish a registry for alzheimer's disease; DSST: digit symbol substitution test.

*Note*: Model 1 adjusted for sex, race, age, income level, and body mass index.

Model 2 adjusted for sex, race, age, income level, body mass index, hypertension, diabetes, coronary heart disease, and stroke.

After excluding individuals only prescribed gabapentin and adjusting for multiple potential confounders, a significant association between malignant tumors and AD remained (aOR: 1.64; 95% CI: 1.00–2.70; *p* = 0.049; Supplementary Table 3).

## 4 Discussion

We detected a risk association between malignant tumors and AD in NHANES data, with NfL serving as an objective measure confirming these findings. Among different tumor types, colorectal and prostate cancers were associated with increased AD risk. Further, patients with malignant tumors aged < 65 years and those in middle–high‐income groups had increased AD risk.

Detecting the onset of AD is challenging, as brain changes can precede clinical symptoms by decades (Wang et al. 2024; Jack et al. 2013). Existing studies show that malignant tumors can influence neuroimaging biomarkers, potentially increasing AD risk (Wang et al. 2024). Treatment of malignant tumors also may impact AD risk. Cranial radiotherapy, often required for certain malignant tumors, has the potential to cause neuronal damage (Lunsford and Kondziolka 2001). Chemotherapy in particular can alter brain structure and function, leading to persistent cognitive impairment (Seigers and Fardell 2011). In an observational investigation of 31 breast cancer patients receiving chemotherapy, 25% experienced a decline in cognitive function (Hurria et al. 2006). Our current research further demonstrates an association between malignant tumors and AD risk.

Our study identified a risk association of AD with both colorectal cancer and prostate cancer. Colorectal cancer and AD not only share common risk genes but also both involve chronic inflammation (Du et al. 2025; Akter et al. 2021; Levy Nogueira et al. 2015). Research shows that an imbalance in the intestinal microbiota of patients can affect neurodegenerative changes via the intestine–brain axis (Kowalski and Mulak 2019; Gagnière et al. 2016). Prostate cancer, the most common malignant tumor among men worldwide, can also lead to neurocognitive disorders due to long‐term androgen derivation therapy (Achard et al. 2021). Patients undergoing this therapy have a 70% higher likelihood of experiencing cognitive issues within 6 months compared to a control group, and twice the risk within 12 months (Gonzalez et al. 2015).

NfL levels are directly proportional to the severity of neurodegenerative lesions (Gaetani et al. 2019). When malignant tumors metastasize, alterations in the tumor microenvironment and damage to host tissues result in increased NfL levels (Kim et al. 2024). Additionally, paraneoplastic syndromes triggered by certain malignant tumors and chemotherapy drugs can cause neuropathy, further increasing NfL levels (Constantinescu et al. 2017; Wang et al. 2025). Our study also found that NfL levels revealed a risk association between malignant tumors and AD. In contrast to objective quantification of NfL, composite cognitive function scores have substantial limitations in statistical validity. These limitations arise from biases inherent in self‐reporting, cultural and educational heterogeneity, and variability in scoring, which collectively reduce the statistical strength of the association between malignancies and AD. Thus, our results with NfL levels strengthen the confidence in reported associations.

Although previous studies suggest a negative association between malignant tumors and AD (Driver et al. 2012; Musicco et al. 2013), these conclusions should be viewed with prudence due to several methodological constraints. First, there is heterogeneity in research design. For instance, Sherzai et al. (Sherzai et al. 2020) observed that patients with prostate cancer had a lower probability of developing AD. However, bidirectional two‐sample Mendelian randomization analysis showed no causal relationship between prostate cancer and AD in European populations (Li et al. 2024). Second, issues related to confounder adjustment and survivorship bias complicate interpretation of findings. These factors prevent accurate control of covariates, such as the survival period of patients with malignant tumors, which may affect study outcomes (Driver 2014; Ospina‐Romero et al. 2020). When using an appropriate normative model that accounts for mortality rate, malignant tumors do not appear to exert a protective effect against AD (Hanson et al. 2017). Further, long‐term malignant tumor survivors (≥ 10 years) have an increased risk of AD mortality compared to the general population (Abdel‐Rahman 2020). Finally, limitations in statistical power and causal inference remain significant challenges. While some studies have used more robust cohort study designs to assess causality, their conclusions may still be biased due to insufficient sample sizes that fail to ensure reliability (Driver et al. 2012; White et al. 2013). As such, current evidence is insufficient to definitively establish a protective relationship between malignant tumors and AD.

In the pathological processes of AD, amyloid‐β induces secretion of p‐tau, leading to tau aggregation. Notably, sex‐related differences have been observed in this pathway, with females exhibiting a stronger association with this process (Biel et al. 2025). In patients with malignant tumors < 65 years old, their nervous systems have not yet undergone physiological aging. Moreover, they are more likely to undergo radiotherapy and chemotherapy, and the neurotoxicity caused by these treatments can directly damage cognitive function, strengthening the association between malignant tumors and cognitive impairment (Kangas et al. 2012; Ouimet et al. 2009). However, in older patients, physiological aging and multiple other factors, such as comorbidities, vascular dementia, and depression may interact to make the risk association between malignant tumors and AD difficult to identify.

Middle‐aged women, who often experience concurrent endocrine changes, represent a key at‐risk population for developing AD (Udeh‐Momoh and Watermeyer 2021). Middle‐ and high‐income groups typically have better medical resources, including regular cognitive screening. Further, long‐term exposure to stress is a well‐established risk factor for AD development (Escher et al. 2019). Since individuals in middle‐ and high‐income brackets often engage in high‐pressure occupations, they may be more prone to develop AD.

### 4.1 Strengths and Limitations

Strengths of this study include the use of multiple NHANES data cycles, which are widely recognized for large sample size and authoritative data, thereby enhancing the reliability of the findings. This study also effectively addresses potential confounding by concurrently assessing both malignant tumors and AD status in participants, which allows for control of mortality‐related biases. Additionally, use of NfL for sensitivity analysis and the separate examination of risk associations between different malignant tumor types and AD further strengthens the generalizability of the conclusions.

Nonetheless, there are some limitations of this study. (1) A key limitation is this study's cross‐sectional structure, which makes it impossible to determine a causal relationship. Future research should use large‐scale, long‐term cohort studies to further investigate the causal relationship between malignant tumors and AD. (2) Although cross‐sectional study designs can mitigate potential survival bias, they cannot eliminate it fundamentally. (3) Diagnosis of malignant tumors in participants was based on self‐reported questionnaire data, while AD diagnosis was determined from prescription records, which may not fully capture participants' actual disease state. (4) Given that we did not apply NHANES‐specific sampling weights, our findings may not be directly generalizable to the broader US population. (5) Although NfL was incorporated into sensitivity analyses, only a single cycle of NfL data was available for evaluation. (6) Unmeasured confounding factors may have influenced the study results. (7) Future studies need to further conduct in vitro and in vivo animal studies to explore deeper molecular associations between malignant tumors and AD risk.

## 5 Conclusions

We detected a significant association between malignant tumors and AD risk, particularly in individuals with colorectal or prostate cancer. The risk of developing AD following a malignant tumor diagnosis was increased in individuals < 65 years old and those in middle‐upper‐income groups. These findings underscore the significance of early AD detection and prevention in patients with a history of malignant tumors.

## Author Contributions

Concept, design and critical revision of the manuscript: Yun Zhang, Yihao Wang, Yang Zhao, Youzhuang Zhu. Acquisition, analysis, or interpretation of data: Yun Zhang, Yang Zhao, Yan Xin, Lei Zhang, Peipei Shan. Drafting of the manuscript: Yun Zhang, Yang Zhao. Supervision: Yihao Wang, Youzhuang Zhu.

## Funding

This trial was supported by the Youth Research Fund of Qingdao University (QDFYQN2023226) and Shandong Provincial Medical and Health Science and Technology Guidance Project(202418000774).

## Peer Review

The peer review history for this article is available at https://doi.org/10.1002/brb3.71066.

## Supporting information




**Supplementary Table**: brb371066‐sup‐0001‐tableS1.docx

**Supplementary Table**: brb371066‐sup‐0001‐tableS2.docx

**Supplementary Table**: brb371066‐sup‐0001‐tableS3.docx
