# Supplementary material for: Associations Between Malignant Tumors and Alzheimer's Disease: A Cross‐Sectional Study
Source: Brain Behav. 2025 Nov 14;15(11):e71066. doi: 10.1002/brb3.71066 (PMC12617259; doi:10.1002/brb3.71066)
Supplement: Supplementary file 1 — Supplementary Table: brb371066‐sup‐0001‐tableS1.docx [file BRB3-15-e71066-s001.docx]

**Supplementary Table 1** Associations among malignant tumors, covariates, and AD

| Variable | OR (95% CI) | *P* | Variable | OR (95% CI) | *P* |
| --- | --- | --- | --- | --- | --- |
| Age |  |  | Depression |  |  |
| <65 years | 1 (reference) |  | Not at all | 1 (reference) |  |
| ≥65 years | 1.76 (1.49–2.07) | <0.001 | Several days | 1.49 (1.22–1.83) | <0.001 |
| Sex |  |  | Most days | 2.42 (1.8–3.26) | <0.001 |
| Male | 1 (reference) |  | Almost every day | 3.85 (2.91–5.09) | <0.001 |
| Female | 1.03 (0.87–1.21) | 0.753 | Hypertension |  |  |
| Body mass index |  |  | No | 1 (reference) |  |
| <30 kg/m^2^ | 1 (reference) |  | Yes | 1.82 (1.54–2.15) | <0.001 |
| ≥30 kg/m^2^ | 1.53 (1.30–1.80) | <0.001 | Diabetes |  |  |
| Race |  |  | No | 1 (reference) |  |
| White | 1 (reference) |  | Yes | 2.57 (2.18–3.04) | <0.001 |
| Black | 0.87 (0.71–1.07) | 0.185 | Coronary heart disease |  |  |
| Hispanic | 0.68 (0.51–0.92) | 0.013 | No | 1 (reference) |  |
| Other | 0.73 (0.59–0.91) | 0.005 | Yes | 1.85 (1.43–2.4) | <0.001 |
| Education |  |  | Stroke |  |  |
| <High school | 1 (reference) |  | No | 1 (reference) |  |
| High school | 0.94 (0.7–1.24) | 0.644 | Yes | 2.64 (2.06–3.37) | <0.001 |
| ≥College | 0.59 (0.44–0.78) | <0.001 | Smoking status |  |  |
| Income level |  |  | Never | 1 (reference) |  |
| Low | 1 (reference) |  | Former | 1.45 (1.21–1.75) | <0.001 |
| Medium | 0.71 (0.59–0.85) | <0.001 | Current | 1.81 (1.47–2.23) | <0.001 |
| High | 0.36 (0.29–0.45) | <0.001 | Cancer |  |  |
| Activity |  |  | No | 1 (reference) |  |
| No | 1 (reference) |  | Yes | 1.55 (1.27–1.89) | <0.001 |
| Yes | 0.8 (0.64–0.99) | 0.042 |  |  |  |

Data are displayed as median (interquartile range) and frequency (percentage).
