# Supplementary material for: Associations Between Malignant Tumors and Alzheimer's Disease: A Cross‐Sectional Study
Source: Brain Behav. 2025 Nov 14;15(11):e71066. doi: 10.1002/brb3.71066 (PMC12617259; doi:10.1002/brb3.71066)
Supplement: Supplementary file 2 — Supplementary Table: brb371066‐sup‐0001‐tableS2.docx [file BRB3-15-e71066-s002.docx]

**Supplementary Table 2** Baseline characteristics of patients with malignant and non-malignant tumors in NfL sensitivity analysis

| Variable | All | Malignant tumors  (n = 138) | Non-malignant tumors (n = 1632) | *P* |
| --- | --- | --- | --- | --- |
| Age |  |  |  | <0.001 |
| <65 years | 1495 (84.5) | 89 (64.5) | 1406 (86.2) |  |
| ≥65 years | 275 (15.5) | 49 (35.5) | 226 (13.8) |  |
| Sex |  |  |  | 0.094 |
| Male | 852 (48.1) | 57 (41.3) | 795 (48.7) |  |
| Female | 918 (51.9) | 81 (58.7) | 837 (51.3) |  |
| Body mass index |  |  |  | 0.725 |
| <30 kg/m^2^ | 1104 (62.4) | 88 (63.8) | 1016 (62.3) |  |
| ≥30 kg/m^2^ | 666 (37.6) | 50 (36.2) | 616 (37.7) |  |
| Race |  |  |  | <0.001 |
| White | 818 (46.2) | 98 (71) | 720 (44.1) |  |
| Black | 315 (17.8) | 16 (11.6) | 299 (18.3) |  |
| Hispanic | 240 (13.6) | 8 (5.8) | 232 (14.2) |  |
| Other | 397 (22.4) | 16 (11.6) | 381 (23.3) |  |
| Education |  |  |  | 0.002 |
| <High school | 107 (6.0) | 6 (4.3) | 101 (6.2) |  |
| High school | 614 (34.7) | 31 (22.5) | 583 (35.7) |  |
| ≥College | 1049 (59.3) | 101 (73.2) | 948 (58.1) |  |
| Income level |  |  |  | 0.007 |
| Low income | 606 (34.2) | 34 (24.6) | 572 (35) |  |
| Medium income | 594 (33.6) | 44 (31.9) | 550 (33.7) |  |
| High income | 570 (32.2) | 60 (43.5) | 510 (31.2) |  |
| Depression |  |  |  | 0.171 |
| Not at all | 1329 (75.1) | 97 (70.3) | 1232 (75.5) |  |
| Several days | 298 (16.8) | 31 (22.5) | 267 (16.4) |  |
| Most days | 72 (4.1) | 3 (2.2) | 69 (4.2) |  |
| Almost every day | 71 (4.0) | 7 (5.1) | 64 (3.9) |  |
| Activity |  |  |  | 0.940 |
| Yes | 457 (25.8) | 36 (26.1) | 421 (25.8) |  |
| No | 1313 (74.2) | 102 (73.9) | 1211 (74.2) |  |
| Hypertension |  |  |  | <0.001 |
| Yes | 510 (28.8) | 69 (50) | 441 (27) |  |
| No | 1260 (71.2) | 69 (50) | 1191 (73) |  |
| Diabetes |  |  |  | <0.001 |
| Yes | 198 (11.2) | 29 (21) | 169 (10.4) |  |
| No | 1572 (88.8) | 109 (79) | 1463 (89.6) |  |
| Stroke |  |  |  | <0.001 |
| Yes | 47 (2.7) | 11 (8) | 36 (2.2) |  |
| No | 1723 (97.3) | 127 (92) | 1596 (97.8) |  |
| Smoking status |  |  |  | 0.010 |
| Never | 986 (55.7) | 61 (44.2) | 925 (56.7) |  |
| Former | 405 (22.9) | 44 (31.9) | 361 (22.1) |  |
| Current | 379 (21.4) | 33 (23.9) | 346 (21.2) |  |

Data are displayed as median (interquartile range) and frequency (percentage).
