# Supplementary material for: Associations Between Malignant Tumors and Alzheimer's Disease: A Cross‐Sectional Study
Source: Brain Behav. 2025 Nov 14;15(11):e71066. doi: 10.1002/brb3.71066 (PMC12617259; doi:10.1002/brb3.71066)
Supplement: Supplementary file 3 — Supplementary Table: brb371066‐sup‐0001‐tableS3.docx [file BRB3-15-e71066-s003.docx]

**Supplementary Table 3** Risk association between malignant tumors and AD (excluding individuals only prescribed gabapentin)

| Variable | OR (95% CI) | | | | | | |
| --- | --- | --- | --- | --- | --- | --- | --- |
|  | n | Crude | *P* | Model 1 | *P* | Model 2 | *P* |
| Malignant tumors,  n (%) |  |  |  |  |  |  |  |
| No | 7842 | 1 (reference) |  | 1 (reference) |  | 1 (reference) |  |
| Yes | 1432 | 1.84 (1.14–2.96) | 0.012 | 1.66 (1.01–2.73) | 0.044 | 1.64 (1.00–2.70) | 0.049 |

Model 1 adjusted for covariates including age, sex, body mass index, race, education, income level, depression, activity, and smoking status.

Model 2 adjusted for covariates including age, sex, body mass index, race, education, income level, depression, activity, smoking status, hypertension, diabetes, coronary heart disease, and stroke.
